# Dietary Control of Inflammation and Resolution

**DOI:** 10.3389/fnut.2021.709435

**Published:** 2021-08-10

**Authors:** Barry Sears, Asish K. Saha

**Affiliations:** Inflammation Research Foundation, Peabody, MA, United States

**Keywords:** 5' adenosine monophosphate-activated protein kinase, SPMs, calorie-restriction, anti-inflammatory diet, omega-3 fatty acids, resolution response, polyphenols, inflammation

## Abstract

The healing of any injury requires a dynamic balance of initiation and resolution of inflammation. This hypothesis-generating review presents an overview of the various nutrients that can act as signaling agents to modify the metabolic responses essential for the optimal healing of injury-induced inflammation. In this hypothesis-generating review, we describe a defined nutritional program consisting of an integrated interaction of a calorie-restricted anti-inflammatory diet coupled with adequate levels of omega-3 fatty acids and sufficient levels of dietary polyphenols that can be used in clinical trials to treat conditions associated with insulin resistance. Each dietary intervention works in an orchestrated systems-based approach to reduce, resolve, and repair the tissue damage caused by any inflammation-inducing injury. The orchestration of these specific nutrients and their signaling metabolites to facilitate healing is termed the Resolution Response. The final stage of the Resolution Response is the activation of intracellular 5' adenosine monophosphate-activated protein kinase (AMPK), which is necessary to repair tissue damaged by the initial injury-induced inflammation. The dietary optimization of the Resolution Response can be personalized to the individual by using standard blood markers. Once each of those markers is in their appropriate ranges, activation of intracellular AMPK will be facilitated. Finally, we outline how the resulting activation of AMPK will affect a diverse number of other intercellular signaling systems leading to an extended healthspan.

## Introduction

There are two distinct phases to the body's response to any injury: the initiation of inflammation and its resolution. Although the molecular biology of the initiation of inflammation is well-understood, the detailed knowledge of the molecular biology of the resolution of inflammation remains an emerging field (1). Successful healing of any inflammation-induced injury requires the coordinated reduction of the initial acute inflammation, followed by resolution of any residual inflammation, and finally repairing the damaged tissue leading to a return to homeostasis. We term this complex process that leads to healing as the Resolution Response (2).

If the Resolution Response is not sufficiently robust to address the initial acute inflammation induced by an injury, there will be a build-up of chronic low-level unresolved inflammation. Although this type of resulting unresolved inflammation is below the perception of pain, it is also a major driving factor for developing a wide variety of age-related chronic diseases, including diabetes, cardiovascular disease, cancer, auto-immune and neurological conditions (2).

The Resolution Response is an evolutionarily conserved mechanism to protect the organism from unresolved injury-induced inflammation. Although microbial invasions will generate an inflammatory response, there are a far greater number of other potential causes of injury-induced inflammation, as shown in Table 1.

**Table 1:** Causes of injury induced inflammation.

Microbial invasions
Physical injuries (internal and external)
Diet-induced
Oxidative stress-induced
Surgery-induced
Drug-induced (cancer drugs in particular)
Stressor-induced (physical, emotional, and environmental)

While there are diverse types of injuries that can induce an initial inflammatory response, the individual components of the Resolution Response that control the healing of the damage are governed by ancient and highly conserved mechanisms under robust dietary control.

The Resolution Response's molecular components that can be directly affected by the diet can be divided into two separate broad classes of signaling agents. One class of signaling agents is hormones consisting of eicosanoids and specialized pro-resolving mediators or SPMs. Eicosanoids would include prostaglandins and leukotrienes, whereas SPMs would include resolvins, protectins, and maresins. The other class of signaling agents is gene modulators. These gene modulators include NF-κB, the gene transcription factor central to the initiation of inflammation, and 5'-adenosine monophosphate-activated protein kinase (AMPK), the master switch of metabolism. AMPK reduces not only NF-κB activity but also is essential to repair damaged tissue.

**Figure 1:**
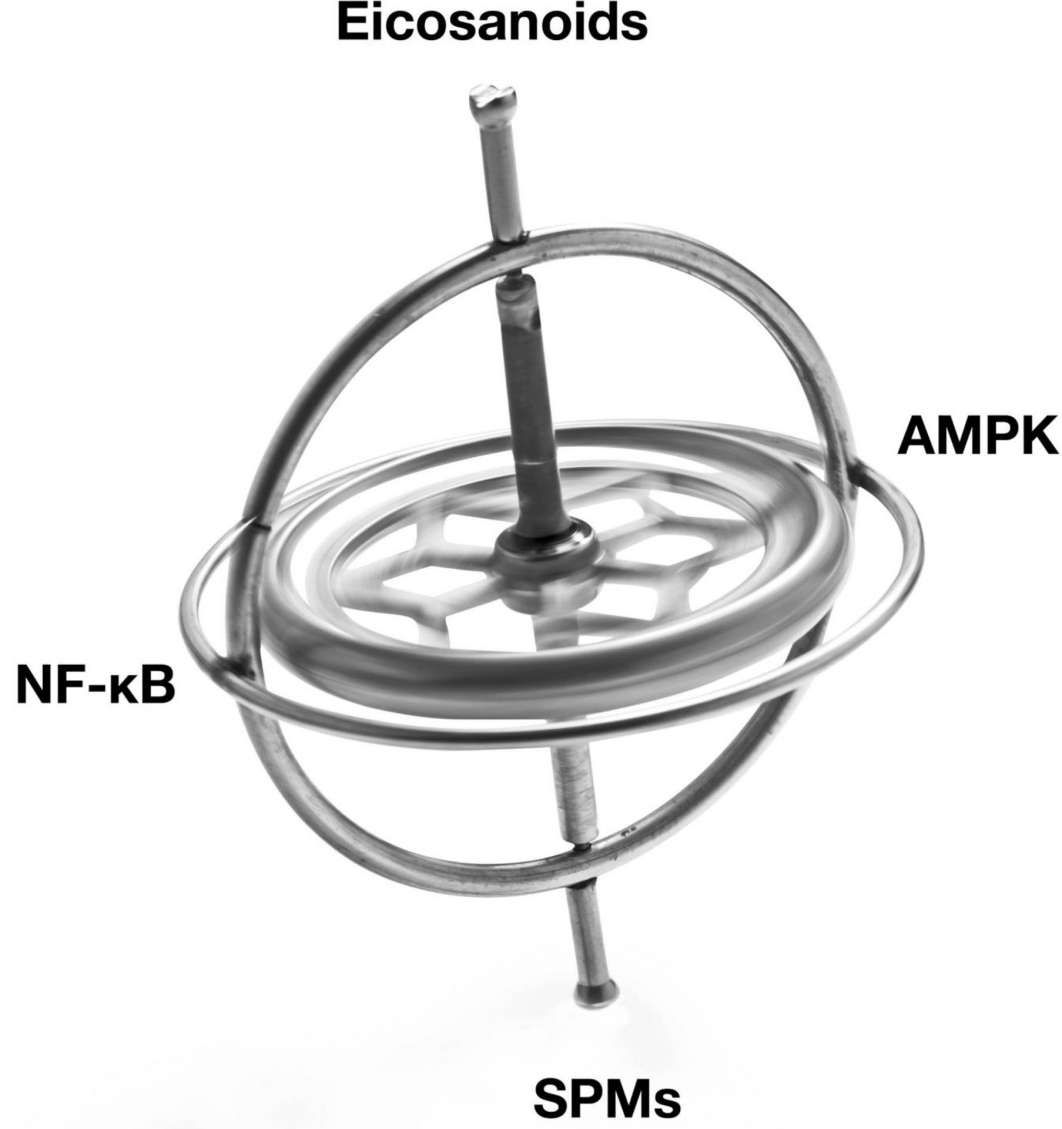
Illustration of the balancing of the signaling agents involved in the Resolution Response. AMPK, 5' adenosine monophosphate-activated protein kinase; NF-κB, Nuclear factor kappa-B; SPMs, Specialized pro-resolving meditators.

A graphical depiction of these signaling agents is shown in Figure 1.

These signaling agents of the Resolution Response represent on-demand responses and must be continually balanced to maintain homeostasis. We use the image of a gyroscope to reflect this need for dynamic balance to maintain dynamic flexibility to switch from inflammation to resolution. Over activation or excessive inhibition of one particular signaling system in Figure 1 may have unintended consequences for the other linked systems. As described later, the ranges of specific blood markers can be altered by the diet to maintain these cellular signaling systems in equilibrium.

Furthermore, acute inflammation is only activated by injury, and it is the onset of inflammation that triggers the start of resolution. The initial acute inflammatory response is protective as it alerts the immune system to respond to the injury. However, if the initial inflammatory response is unresolved, this leads to chronic low-level inflammation. This unresolved inflammation results in tissue damage that transforms the otherwise protective initial inflammatory response associated with many chronic disease conditions. To successfully heal from any injury-induced inflammation, one must increase those diet-controlled nutrients or their metabolites to activate AMPK and enhance SPM formation to address unresolved inflammation. Simultaneously, one also has to decrease the intake of those diet-controlled nutrients that can promote excessive NF-κB activity and increased eicosanoid formation. Thus, rather than concentrating on any single dietary component of the Resolution Response, one must focus on the broader vision of maintaining all of the diet-controlled factors of the Resolution Response within appropriate operating ranges. Thus, the Resolution Response can be best understood from a dynamic systems-based biology viewpoint consisting of complex, interrelated systems necessary for successful healing.

It should be pointed out that the role of vitamins and minerals in the immune response is relatively minor (3, 4). The Resolution Response is dependent on how specific dietary factors are important in the generation of either signaling hormones (eicosanoids and SPMs) or the control of intracellular factors (NF-κB and AMPK) are far more critical in controlling the immune response.

### General Description of the Dietary Impact on Each Phase of the Resolution Response

**Figure 2:**
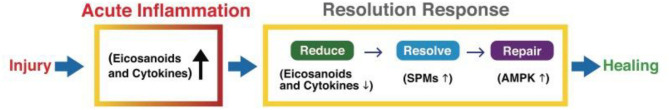
A graphic illustration of the sequential events for a successful Resolution Response to injury-induced inflammation. AMPK, 5' adenosine monophosphate-activated protein kinase; SPMs, Specialized pro-resolving meditators.

The three distinct phases of the Resolution Response can be summarized as (a) reducing injury-induced inflammation, (b) resolving residual inflammation, and (c) repairing the tissue damage caused by injury-induced inflammation, as shown in Figure 2.

It should be noted that both acute inflammation and the Resolution Response are quiescence systems that are only activated by injuries that induce inflammation.

**Figure 3:**
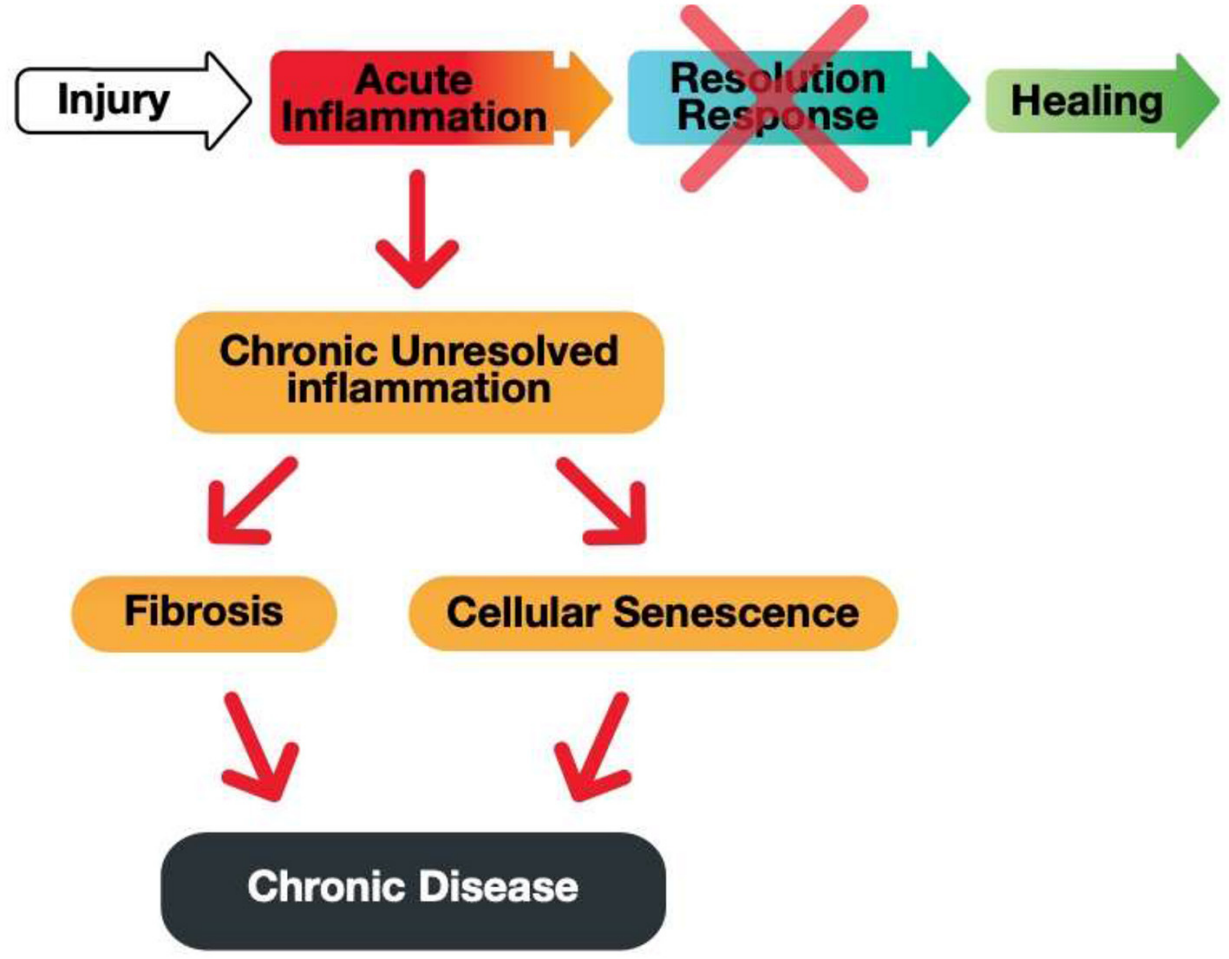
A graphic illustration of the consequences of a blocked Resolution Response preventing healing of an injury-induced inflammation.

Acute inflammation is critical for proper immunological function. However, an equally robust Resolution Response must counter acute inflammation to prevent the build-up of unresolved inflammation that can lead to either fibrosis or cellular senescence, as shown in Figure 3.

### Reducing Inflammation

A highly effective way to reduce existing inflammation is following a highly defined anti-inflammatory diet. The problem is how to describe such a diet.

The most important consideration for any anti-inflammatory diet is calorie restriction. Any reduction of excess calorie intake will lead to a decrease in systemic oxidative stress. Calorie restriction has been the most successful therapeutic intervention to improve healthspan (defined as longevity minus years of disability) in virtually every species studied (5). Significant metabolic benefits have been achieved by calorie restriction in healthy overweight and normal-weight individuals who participated in the various CALERIE (Comprehensive Assessment of the Long-Term Effects of Reducing Intake of Energy) studies (6, 7).

Successful lifetime calorie restriction depends on the ability of such a diet to increase satiety. From this perspective, consuming adequate protein levels at each meal may represent a necessary first step. This concept is known as protein leveraging (8, 9). Potential protein leveraging mechanisms may include increasing glucagon levels to stabilize blood glucose levels in the blood (10) and the increased release of satiety hormones such as PYY and GLP-1 from the gut (11). In addition, the intake of fermentable fiber in a calorie-restricted diet is also essential for generating short-chain fatty acids (SCFA) that further enhance the signaling intensity of PYY and GLP-1 generated by the protein intake at a meal (12).

An anti-inflammatory diet should also substantially reduce the omega-6 fatty acid arachidonic acid (AA) levels in the plasma membrane. AA is the primary building block of eicosanoids. The vast majority of eicosanoids derived from AA are pro-inflammatory hormones that can significantly intensify any initial inflammatory response, making it more challenging to resolve the initial acute inflammation. However, a specific AA level in the plasma membrane is necessary to generate the eicosanoids required to create an acute inflammatory response. This process begins with the activation of phospholipase A_2_ that releases AA from the plasma membrane phospholipids. This free AA is immediately metabolized into eicosanoids. However, excess AA levels in the plasma membrane will cause increased amplification of the initial inflammatory response. Although reducing the dietary intake of AA is one possible way to achieve this goal, it should be emphasized that much of the AA in the body (and especially in the plasma membrane) comes from the metabolism of the linoleic acid into AA (13). The metabolic conversion of linoleic acid to AA is accelerated by elevated insulin levels generated either by a consistently high glycemic load of the diet or by existing insulin resistance. In either case, elevated insulin levels will activate the rate-limiting enzymes (delta-6-desaturase and delta-5-desaturase), leading to increased AA formation from dietary intake of linoleic acid (14).

On the other hand, the hormone glucagon, induced by the diet's protein content, will inhibit the same desaturase enzymes (14, 15). Thus, the balance of the protein-to-glycemic load of an anti-inflammatory diet is essential in reducing excess formation of AA that can result in excessive eicosanoid-driven inflammation (13). Thus, a primary requirement of an anti-inflammatory diet is to be low in linoleic acid, which will further decrease AA levels by the above-mentioned metabolic pathways.

The development of insulin resistance can also increase AA formation. Insulin resistance appears to be strongly influenced by cytokine levels (especially TNFα) induced by activation of NF-κB (16–18).

NF-κB is activated by high levels of saturated fatty acids (primarily palmitic acid) in the blood that can interact with the toll-like receptors TLR-2 and TLR-4. Activation of NF-κB results in the increased production of cytokines (19, 20). Thus, an additional dietary requirement for an anti-inflammatory diet is a low intake of saturated fats, especially palmitic acid.

Increased insulin resistance eventually leads to elevated blood glucose levels that cause beta-cell function in the pancreas to begin to fail (21). The combination of high blood glucose levels and oxidative stress generated by excess calorie intake can increase Advanced Glycosylated End products or AGE (22). These glycosylated proteins can interact with specific receptors (RAGE) on the cell surface, providing another diet-related pathway to increase cytokine production by activating NF-κB (23).

Just as it is necessary to reduce the dietary intake of omega-6 and saturated fatty acids, it is also essential to increase the omega-3 fatty acid intake, especially of the long-chain omega-3 fatty acids eicosapentaenoic acid (EPA) and docosahexaenoic acid (DHA). EPA is a feedback inhibitor of the delta-5-desaturase enzyme that is the rate-limiting step in the production of AA (24). Thus, the higher the levels of EPA in the diet, the less AA is generated.

Eicosanoids can be generated from EPA, but not DHA. The eicosanoids generated from the EPA are ~100–1,000 times lower in their inflammatory intensity than the same eicosanoids derived from AA (25). Thus, the eicosanoids derived from EPA are not strictly anti-inflammatory hormones, but they are far weaker pro-inflammatory hormones than those derived from AA. The net result is a reduction in the intensity of the inflammatory response.

Another reason for increasing the omega-3 levels in an anti-inflammatory diet is the reduction of inflammasome activation. Inflammasomes are intracellular structures formed in response to microbe-derived pathogen-associated molecular patterns (PAMPs) or danger-associated molecular patterns (DAMPs) sensed within the cell (26). Once activated, inflammasomes will cause the generation of pro-inflammatory cytokines such as IL-1β and IL-18 (27). The NLRP3 inflammasome is the most investigated of the various inflammasomes (28). Omega-3 fatty acids such as EPA and DHA can inhibit the activation of inflammasomes (29). However, it appears that DHA may be more effective than EPA in this regard (30).

Finally, an anti-inflammatory diet should reduce the inflammatory effects of metabolic endotoxemia by strengthening the mucosal barrier in the gut (31). Improvement in the integrity of the gut's tight junctions can result from the increased levels of the bacterium *Akkermansia muciniphila*. The population of this bacterium in the gut can be enhanced with an increased intake of fermentable fiber, omega-3 fatty acids, and polyphenols (32, 33).

The dietary foundation for reducing diet-induced inflammation would consist of the following nutritional composition: calorie restriction with adequate protein coupled with a moderate level of low glycemic-load carbohydrates to reduce excess glucose intake. Furthermore, the diet should be low in total fat (especially omega-6 and saturated fatty acids), yet with sufficient levels of fermentable fiber, omega-3 fatty acids, and polyphenols.

### Increasing Resolution

Reducing the inflammation caused by an injury is only the first step toward the ultimate healing of any tissue damage. The second obligatory step of the Resolution Response is resolving any residual inflammation (34). Unlike the variety of dietary interventions that help reduce inflammation, increasing resolution of any residual inflammation is purely a function of omega-3 fatty acids in the diet that are the building blocks to produce levels of adequate Specialized Pro-Resolving Mediators (SPMs). SPMs are hormones that control the resolution of residual inflammation. SPMs represent a diverse superfamily of hormones consisting of three primary subfamilies: resolvins, maresins, and protectins. These SPMs are biosynthesized from EPA, DHA, and docosapentaenoic acid (DPA) (35, 36).

SPMs are critical for several distinct stages of resolution, including (a) stopping neutrophil swarming to the injury site, (b) causing the transition of pro-inflammatory macrophages (M1) to pro-resolution macrophages (M2) to remove cell debris from the injury site, and (c) increasing efferocytosis to remove apoptotic cells (37). In addition, the production of SPMs may also be a critical factor in preventing the priming of the inflammasome (38, 39).

The levels of omega-3 fatty acids required to achieve these goals can be estimated by the ratio of leukotrienes to SPMs (40, 41). Unfortunately, such a determination requires highly sophisticated instrumentation. However, leukotrienes are derived from AA, and many SPMs (such as the E-series resolvins) are derived from EPA. Consequently, the ratio of AA/EPA in the blood can serve as an upstream surrogate marker to determine whether therapeutic levels of omega-3 fatty acids are present to generate sufficient levels of SPMs to complete the resolution phase of the Resolution Response.

### Altering Gene Expression

The final and most complex phase of the Resolution Response is activating the master switch of metabolism, AMPK (2, 42). AMPK is a highly conserved energy sensor controlled by the balance of AMP and ATP levels in a cell. As the AMP/ATP ratio increases as occurs with calorie restriction, AMPK is activated, which sets in motion the phosphorylation of a broad cascade of gene transcription factors that switch metabolism from anabolic to catabolic to restore ATP levels (43–46).

**Figure 4:**
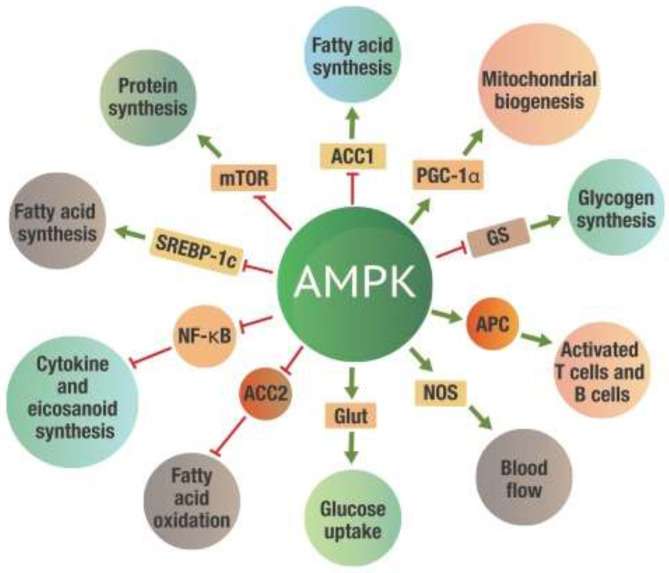
Metabolic effects of AMPK activation. Green arrows on the “spokes” indicate activation, red lines with a bar at the end indicate inhibition. ACC 1 and ACC2, -Acetyl-CoA carboxylase 1 and 2; AMPK, 5' adenosine monophosphate-activated protein kinase; Glut, Glucose transporter protein; GS, Glycogen synthetase; mTOR, mammalian target of rapamycin; NF-κB, Nuclear factor kappa-B; NOS, Nitrogen oxide synthetase; PGC-1α, Peroxisome proliferator-activated receptor gamma coactivator 1-alpha; SREBP-1c, Sterol regulatory element-binding protein 1c.

Figure 4 indicates just a few of the many metabolic effects that take place once AMPK is activated.

While all these actions will significantly affect metabolism, perhaps the most critical benefit of AMPK activation is inhibiting NF-κB activity (47–49). The inhibition of NF-κB leads to a substantial reduction of both excess eicosanoid and cytokine levels. The activation of AMPK thus reduces inflammation induced by NF-κB activation. This metabolic control of NF-κB by AMPK is critical for the successful repair of damaged tissue.

This repair process starts with increased autophagy to supply the molecular building blocks for tissue repair and increased mitophagy to replace damaged mitochondria to provide the energy required for tissue repair (50). These processes are controlled by AMPK *via* activation of ULK-1, which is the first step to increase mitophagy (50).

A potential link between increased SPM formation and increased AMPK activity appears to be mediated by receptors for various SPMs. One well-characterized receptor is FPR2/ALX, a receptor for lipoxin A_4_ and the anti-inflammatory/pro-resolution protein annexin (51). FPR2/ALX is also a receptor for the Resolvin D1 (RvD1) derived from DHA (52). Thus, it is very likely that RvD1 and other SPMs, signaling through similar receptors, may also be instrumental for increased AMPK activation (53–55).

**Figure 5:**
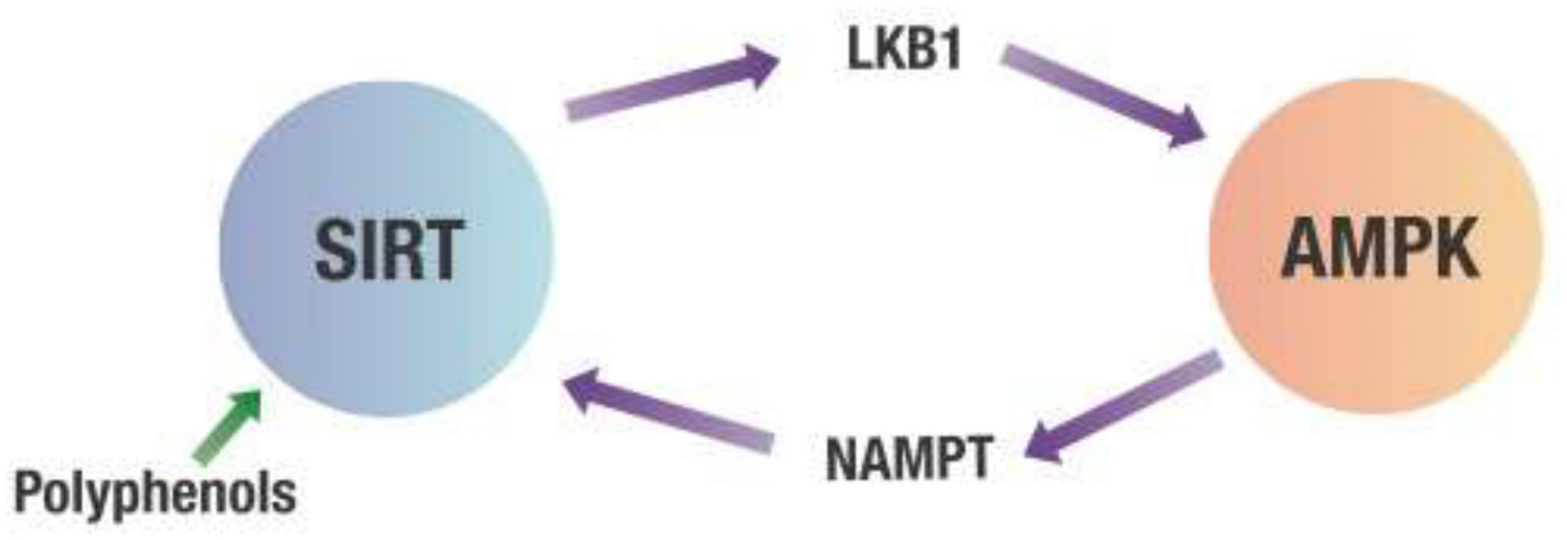
Crosstalk between SIRT and AMPK. Any decrease in the cell's energy state measured by an increased AMP/ATP ratio will activate AMPK. This activation of AMPK leads to increasing NAMPT activity that produces NAD^+^ required for SIRT deacetylation activity. SIRT then deacetylates LKB1, which activates AMPK. AMPK, 5' adenosine monophosphate-activated protein kinase; LKB1, Liver kinase B1; NAMPT, Nicotinamide phosphoribosyltransferase; SIRT, Sirtuins.

However, the most potent dietary effector of AMPK may be polyphenols. Polyphenols activate AMPK indirectly by binding to various sirtuins (SIRT) which are deacetylating enzymes dependent on NAD^+^ (56, 57). One of the multiple targets for SIRT is liver kinase B1 (LKB1). Once LKB1 is deacetylated, it activates AMPK, which inhibits NF-κB (58, 59). In addition, AMPK activates the rate-limiting enzyme (nicotinamide phosphoribosyltransferase or NAMPT) of the salvage pathway that regenerates NAD^+^, needed for the deacetylating activity of various SIRT proteins. This crosstalk between SIRT and AMPK creates a positive feedback loop for AMPK activation (60–64). This is shown in Figure 5.

The only problem with dietary polyphenols as AMPK activators is their limited water-solubility. However, specific subclasses of polyphenols such as anthocyanins (particularly delphinidins) have high water-solubility making it possible to obtain adequate blood levels to increase AMPK activity (65–69).

AMPK activity is under robust dietary control activated by calorie restriction, SPMs, and polyphenols. On the other hand, AMPK activity is inhibited by excess calorie intake and elevated blood glucose levels (70, 71). Thus, one can obtain the maximum dietary activation of AMPK activity by following a calorie-restricted diet with a low glycemic index with adequate omega-3 fatty acids and water-soluble polyphenols such as anthocyanins, especially bioavailable delphinidins (72, 73).

### Specific Dietary Guidelines to Optimize the Resolution Response

Optimizing the Resolution Response requires viewing dietary nutrients as signaling agents, as shown in Table 2.

**Table 2:** Dietary nutrients and their signaling agents in the resolution response.

**Nutrient**	**Signaling agent**
Protein	Glucagon, PYY, and GLP-1
Carbohydrates	Insulin
Fats	Eicosanoids and SPMs
Fermentable fiber	Short-chain fatty acids
Polyphenols	AMPK

### Protein

Adequate protein intake plays a critical role in the long-term adherence of calorie restriction to activate AMPK. Sufficient protein levels at every meal are necessary to control satiety *via* protein leveraging (9, 74). Protein leveraging is based upon the hypothesis that the protein levels at each meal determine the level of appetite suppression of that meal. Without adequate appetite suppression between meals, long-term success for calorie restriction is highly unlikely. There are two potential mechanisms of protein leveraging. The first mechanism is to have adequate dietary protein at any meal to release sufficient levels of hormones such as PYY and GLP-1 from the small intestine that goes directly to the brain *via* the vagal nerve to reach the hypothalamus to reduce hunger (75, 76). Thus, the less protein consumed in a meal, the more likely additional calories will be needed to be consumed at that meal to cause sufficient appetite suppression by alternative pathways. The second mechanism is the increase in glucagon levels in the blood stimulated by dietary protein (10). Glucagon will release stored glycogen from the liver to maintain stable blood glucose levels, thereby reducing hunger. This outcome of improved hunger control is also suggested by a recent study demonstrating that the postprandial glycemic dip at 2–3 h predicted future appetite and energy intake (77).

Clinical data suggest that weight regain after controlled purposeful weight loss is reduced with a higher protein percentage in the diet. It appears that ~25% of the total calories as protein at each meal may be a threshold for this effect (10, 78). For a 400-calorie meal, this would mean consuming ~25 g of protein. However, the daily dietary protein required is based on the individual's lean body mass and physical activity (13). For the average female, this will be ~75 g of protein per day. For the average male, the protein level will be about 100 g of protein per day.

It should be noted that these protein intake levels for an anti-inflammatory diet are typical for the US population (79). Although these recommended daily protein levels are slightly higher than the generally recommended minimum daily protein intake, they are necessary because calorie restriction for an anti-inflammatory diet can reduce lean body mass (80). Furthermore, the total protein should be spread evenly throughout the day for improved hormonal control (13, 81).

Another nuance of protein leveraging is the timing of protein consumption during a meal. Eating protein before consuming carbohydrates generates more significant glycemic control in type 2 diabetic subjects than consuming carbohydrates first before protein (82, 83). One potential reason may be that protein requires a longer transit time in the small intestine to reach the L-cells that secrete PYY and GLP-1. These gut hormones stimulated by protein go directly *via* the vagal nerve to the brain's appetite center in the hypothalamus to generate satiety. This timing factor suggests that the blood levels of amino acids needed to stimulate glucagon will rise more slowly than glucose levels (especially high-glycemic load carbohydrates rich in glucose) required to stimulate insulin release. Thus, consuming protein first in a meal should generate a more favorable postprandial glucagon-to insulin balance after the meal. Clinical data suggests an improved balance of glucagon-to-insulin leads to a substantial reduction in consumed calories under *ad libitum* conditions after consuming two consecutive balanced meals compared to isocaloric meals with a lower protein-to-carbohydrate ratio (10).

### Carbohydrates

Since excess dietary glucose is an inhibitor of AMPK, the glucose content in a meal and its entry rate into the bloodstream will be essential for optimizing the Resolution Response. The glycemic index measures the rate of entry of glucose in the blood of a defined amount of particular carbohydrate-containing food. However, a more relevant metabolic parameter is the glycemic load. The glycemic load considers how rapidly the total carbohydrate of a meal raises blood glucose levels (84). Meals consisting of a high glycemic load will inhibit AMPK activity. The highest glycemic load comes from having grains and starches as the primary carbohydrates in a meal. Therefore, for minimum inhibition of AMPK, the carbohydrate content of a meal should consist primarily of carbohydrates with a low glycemic impact. These carbohydrates would mainly consist of primarily non-starchy vegetables and limited amounts of fruits (mostly berries). Epidemiology studies have indicated that individuals consuming 10 servings per day of non-starchy vegetables and fruits have lower mortality and morbidity than those consuming fewer servings of these low glycemic impact carbohydrates (85). This epidemiological observation would be consistent with decreased inhibition of AMPK activity.

### Fats

Total fat content should remain low for maintaining calorie restriction. However, one must also consider the composition of that total fat intake. The balance of omega-6 to omega-3 fatty acids should be no >2:1. A lower omega-6 to omega-3 fatty acid ratio will ensure a better balance of their bioactive biosynthetic products (eicosanoids coming from omega-6 fatty acids and SPMs from omega-3 fatty acids) help improve the resolution of any injury-induced inflammation. The omega-6 to omega-3 fatty acid balance in the United States was ~10:1 in 1999 (86, 87). The levels of saturated fats (principally palmitic acid) should also remain low because of their potential generation of inflammation in the hypothalamus (88). Increased hypothalamic inflammation can also potentially attenuate satiety signals from the gut and the blood (89). As discussed earlier, high levels of palmitic acid can interact with the TLR-2 and TLR-4 receptors to activate NF-κB to generate cytokines (19, 20). Thus, the bulk of the limited fat content for an anti-inflammatory diet should come from monounsaturated fatty acids.

### Macronutrient Balance

The macronutrient balance of the diet at every meal can also further control hormonal responses. Ideally, the level of low-glycemic-load carbohydrates should be approximately one-third more than the protein content in a meal (13). Such a protein-to-carbohydrate balance is very similar to that estimated for a Paleolithic diet (90). Furthermore, this balance of protein-to-carbohydrate should be consistent for each meal. It is known from clinical experiments that the protein-to-carbohydrate ratio profoundly affects the blood's resulting insulin-to-glucagon balance under isocaloric conditions (10). This hormonal balance is essential since the delta-6 and delta-5 desaturase enzymes that convert the omega-6 fatty acid linoleic acid into arachidonic acid (AA) are activated by insulin (14). In contrast, glucagon inhibits these same enzymes, decreasing the potential excess formation of AA, thereby reducing possible excess eicosanoid formation (14, 15).

Finally, to maintain appropriate calorie restriction, the total fat content of the diet should be <50 g per day. Thus, the macronutrient composition of an anti-inflammatory diet should be ~1 g of fat for every 2 g of protein and every 3 g of carbohydrate at every meal. This total macronutrient balance is also consistent with estimates of Paleolithic diets (90). Placebo-controlled trials using calorie-restricted diets have demonstrated that the macronutrient balance described above appears to reduce inflammation (91–93) significantly. These differences in the reduction of inflammation based on the protein-to-carbohydrate ratio may be related to the change in the balance of insulin and glucagon observed in different protein-to-carbohydrate ratios seen in cross-over diet studies (10). Changes in the insulin-glucagon balance can affect the activity of various desaturases are critical for preventing excess arachidonic acid production (14). These initial results suggest that the protein-to-carbohydrate balance of a calorie-restricted diet may significantly improve its anti-inflammatory effects.

### Fermentable Fiber

Gut bacteria require the dietary intake of fermentable fiber to generate short-chain fatty acids (SCFA). SCFA act as signaling agents to maintain the gut barrier's integrity by decreasing inflammation in the gut wall (94, 95).

Improving the integrity of the gut wall will reduce metabolic endotoxemia as a significant source of gut-induced inflammation (96). SCFA produced in the gut can also significantly affect neurological function *via* the vagus nerve (97). Finally, SCFA have a significant role in maintaining satiety by enhancing the secretion of PYY and GLP-1 from the gut (98, 99).

### Polyphenols

Polyphenols constitute a large group of more than 8,000 compounds that can be potentially metabolized into less complex phenolic compounds that can also act as signaling agents (100). It is challenging to measure polyphenols in the blood. However, the metabolites of those polyphenols that are absorbed can be found in the urine. Studies have suggested that the increased levels of polyphenols in the urine are associated with reduced frailty and mortality compared to the estimated dietary intake of polyphenols from food diaries (101, 102). Furthermore, only specific polyphenol structures appear maximally active as allosteric agents to increase the sirtuins' deacetylating activity, as described earlier. The polyphenols with the most significant potential to cause an allosteric activation of sirtuins appear to require an intact flavonoid structure and a net positive charge to enhance their water solubility (57). The subgroup of polyphenols that has both structural characteristics is anthocyanins. Berries are a rich source of anthocyanins. It should be noted that increased anthocyanin intake is associated with decreased incidence of cardiovascular disease, although the effect appears to be stronger in women than men (103, 104).

### Vitamins and Minerals

Compared to the above-mentioned dietary factors, the role of vitamins and minerals will have a relatively minor effect on inflammation and resolution and the immune response (3, 4). Furthermore, it has been calculated that a calorie-restricted anti-inflammatory diet described earlier is rich in non-starchy vegetables and limited amounts of fruits and would supply adequate levels of vitamins and minerals (105).

### Clinical Markers to Determine an Optimal Resolution Response

The ultimate approach to healing is to optimize the Resolution Response. Success requires reducing, resolving, and repairing inflammatory damage caused by any injury. This goal can be achieved by reducing the stresses on lipid, glycemic, and inflammatory responses. These various metabolic responses are under significant dietary control. Therefore, the more the stress levels of each of these three metabolic responses are maintained within appropriate ranges, the greater the degree to which the Resolution Response becomes optimized.

Successful dietary management of the Resolution Response can be determined by the extent to which each of the clinical markers of metabolic stress is maintained within their desired ranges. Such blood markers must be highly validated, easily obtained, and provide clear guidelines for personalizing the individual's diet.

The three clinical markers that meet these criteria are the following:

(a) Reducing lipid stress: A primary factor is causing lipid stress is insulin resistance. Therefore, the TG/HDL ratio (measured in mg/dL) should be <1 for controlling insulin resistance (106–113).
(b) Reducing inflammatory stress: Inflammatory stress is caused by an imbalance in the production of eicosanoids and SPMs. The AA/EPA ratio should be maintained between 1.5 and 3 to maintain an appropriate ratio of precursors for a balanced formation of both eicosanoids and SPMs. A significant reduction of various cytokines is observed when this range of the AA/EPA ratio is achieved by appropriate supplementation with omega-3 fatty acids (114–116). The average AA/EPA ratio in the US is >20 (117, 118), indicating the existence of an unfavorable balance of eicosanoids to SPMs.
(c) Reducing glycemic stress: The HbA1c level should be maintained between 4.9 and 5.1 percent, indicating the lack of glucose inhibition of AMPK activity (70).

The recommended appropriate ranges for these markers are lower than the typical average levels for healthy individuals, but they are still within normal ranges. Therefore, only when all three of these three clinical markers are in their appropriate ranges can the Resolution Response be considered to be optimized for an individual.

Furthermore, each of the markers can be modulated by specific dietary interventions. For example, the level of insulin resistance can be significantly reduced by following an anti-inflammatory diet. In particular, the dietary intake of omega-3 fatty acids strongly influences the AA/EPA ratio. Finally, the dietary intake of polyphenols to activate AMPK will strongly affect the HbA1c levels. It should be noted that AMPK can also be activated by increased SPM synthesis and calorie restriction that is the foundation for an anti-inflammatory diet. Thus, there is significant crosstalk of the various dietary components as discussed above.

### Potential Need for Supplementation

In principle, an appropriate anti-inflammatory diet should be sufficient to optimize the Resolution Response. However, the diet will often require supplementation with omega-3 fatty acids and polyphenols to reach the target ranges of the clinical markers described above. This need for potential supplementation is because the levels of omega-3 fatty acids in the blood required to resolve the residual inflammation caused by the injury sufficiently are often beyond the intake provided by the best anti-inflammatory diet. This need for supplementation is also the case for the amounts of polyphenols needed to activate AMPK to repair damaged tissue.

In particular, supplementation with EPA and DHA concentrates may be required to increase the biosynthesis of the SPMs needed to resolve residual inflammation and reduce inflammasome formation (29, 38, 39, 119–121). The best sources of polyphenols to enhance AMPK activity will come from polyphenol concentrates of the anthocyanin family. These polyphenols can enhance the allosteric activation of sirtuins to indirectly activate AMPK (55) and inhibit inflammasome formation (122, 123). Such polyphenol concentrates should also be devoid of glucose that would otherwise impede the activation of AMPK.

The recommended dosage of the above supplements can be obtained from the selected clinical trials. It has been demonstrated that a dose of 2.5 g of EPA and DHA per day for 8 weeks can significantly reduce cytokines levels in elderly obese individuals compared to a placebo group (115). The REDUCE-IT study used 3.9 g of EPA over an average of 4.9-years demonstrated a significantly reduced number of CHD events in subjects with elevated triglycerides and already taking statins compared to the placebo group (124). In patients with coronary artery disease, a daily dose of 3.4 g of EPA and DHA per day for year generated SPMs in the active group, but not the placebo group (125). Thus, it would be recommended that an initial starting supplemental dose of omega-3 fatty acids would be between 2.5 and 4 g per day. The precise level of omega-3 supplementation can be determined from the level required to reduce the AA/EPA ratio in the blood to between 1.5 and 3.

The levels of polyphenols, and in particular anthocyanins, are estimated to be at least −150 to 500 mg per day. An open study using 180 mg of anthocyanins per day demonstrated a statistically significant decrease in the HgA1c levels in prediabetics within 4 weeks that continued to decrease at 12 weeks (126). A placebo-controlled study using 450 mg per day of anthocyanin supplementation produced a statistically significant decrease in oxidative stress after 30 days of supplementation in the smokers in the active group compared to the control group of smokers as measured by decreased isoprostane levels. After anthocyanin supplementation was stopped, the level of isoprostanes in the active group returned to their original levels after 40 days (127).

Reaching the target HbA1c ranges determines the amount of optimal level of polyphenol supplementation that may be required. Since individuals are not genetically identical, the levels of potential supplementation are personalized by the blood markers already described.

While any one of the three dietary interventions is helpful for partial activation of AMPK, we contend that the combination of all three nutritional interventions will be needed to have a significant and sustained effect on AMPK activation for meaningful therapeutic results.

**Figure 6:**
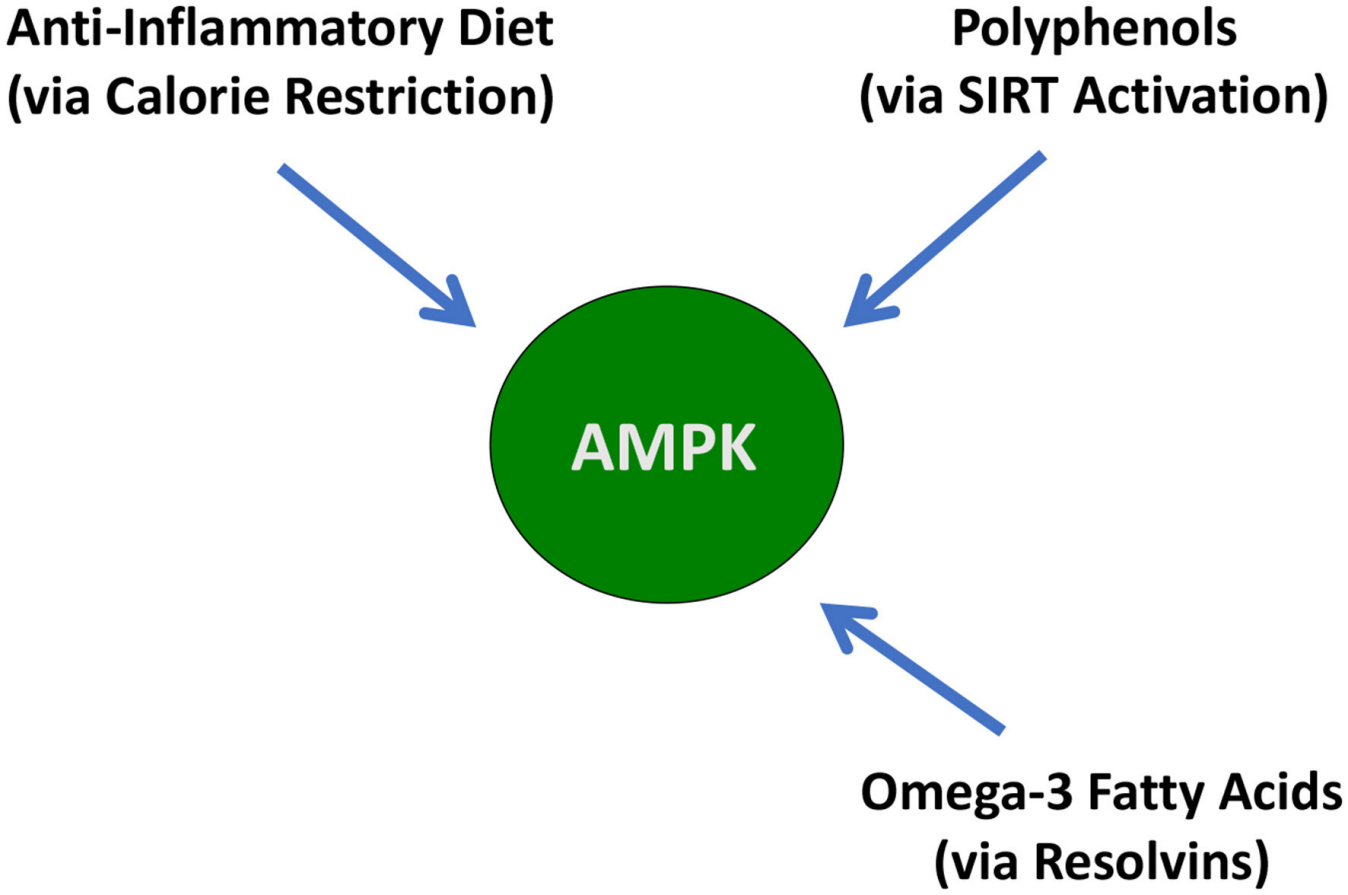
A graphical description of the dietary interventions that can activate AMPK.

This need for multiple dietary interventions for maximum activation of AMPK is shown in Figure 6.

### The Potential Relationship of the Resolution Response to Insulin Resistance

The concept of insulin resistance has been used for more than 80 years (128). Its relationship to a larger group of chronic conditions began to be more recognized by the work of Gerald Reaven (129). However, it still is not clear exactly what causes insulin resistance (130, 131). However, it is known that insulin resistance is also associated with chronic low-level inflammation (132–135). Furthermore, the clinical marker of insulin resistance is hyperinsulinemia. Exactly how hyperinsulinemia causes the wide variety of metabolic disturbances associated with insulin resistance is still open to question (130, 131).

**Figure 7:**
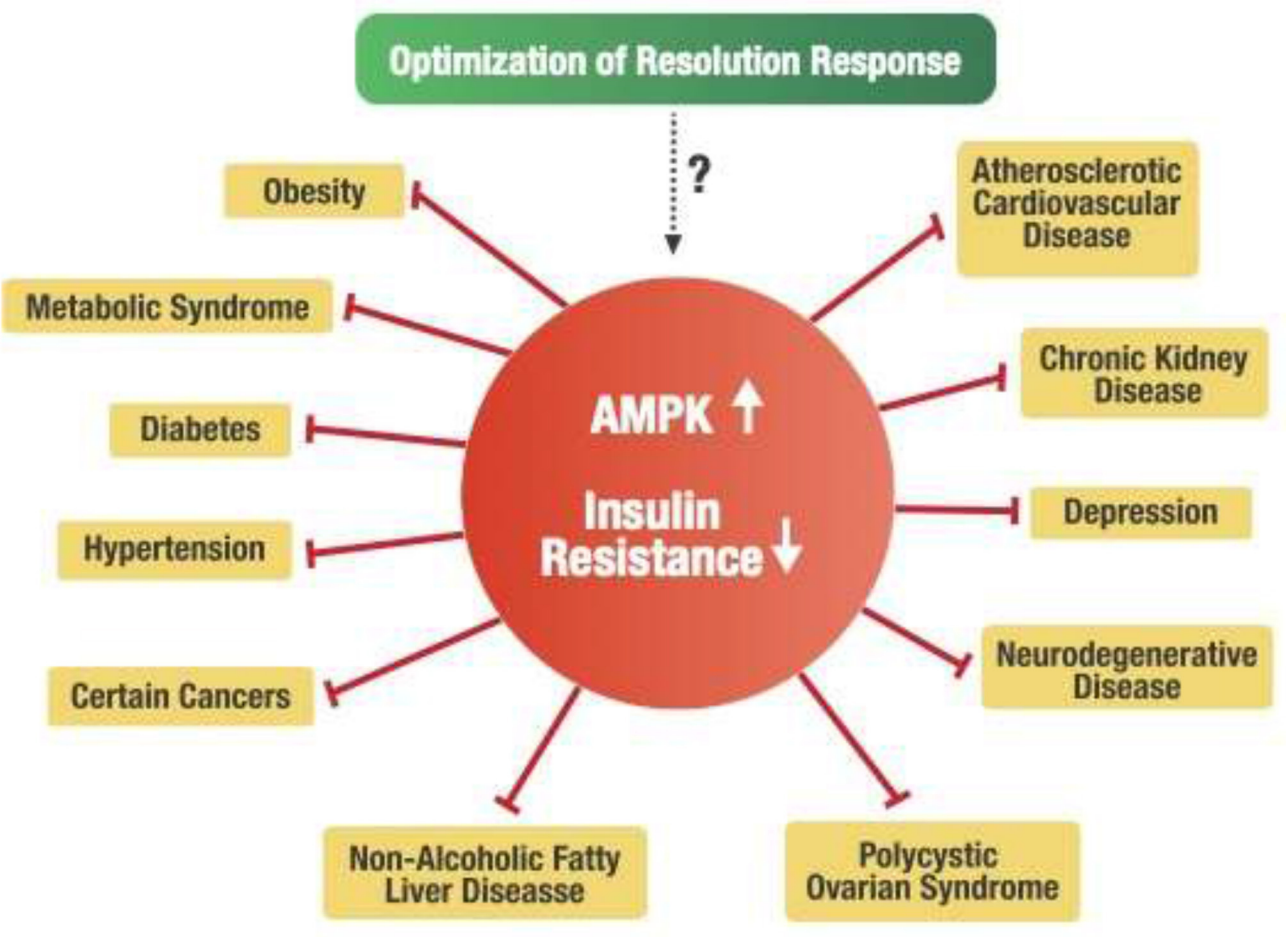
Potential linkage of the Resolution Response to insulin resistance and related chronic disease conditions.

An alternative hypothesis can be formulated that a deficiency of AMPK activity may be the central factor in developing metabolic disturbances within the cell that are associated with insulin resistance. For example, it is known that insulin resistance is strongly associated with reduced AMPK activity (44, 136–138). Consequently, metabolic disorders such as obesity, metabolic syndrome, type 2 diabetes, and non-alcoholic fatty liver disease (NAFLD) strongly associated with insulin resistance have been directly linked to decreased AMPK activity (43, 139, 140). Furthermore, other chronic conditions related to increased insulin resistance include hypertension (141), cardiovascular disease (142), polycystic ovary syndrome (143), chronic kidney disease (144), various types of cancer (145), depression (146), and neurodegenerative diseases such as Alzheimer's and Parkinson's (147). These diverse chronic conditions are also considered pro-inflammatory conditions related to unresolved inflammation caused by blocked Resolution Response leading to deficiency of AMPK activity in target cells. Therefore, maintaining a constant dietary optimization of the Resolution Response may offer a fundamental central dietary approach for potentially managing those many chronic conditions associated with insulin resistance, as shown in Figure 7.

From this perspective, one could potentially redefine insulin resistance as a deficiency of AMPK activation caused by a blocked Resolution Response. It should be noted that the most successful group of drugs for treating insulin resistance have been thiazolidinediones (TZDs).

One hypothesized mode of action of this class of drugs is the activation of AMPK (148–150). Therefore, we feel that optimization of the Resolution Response by the appropriate dietary interventions to increase the activity of AMPK within the cell may potentially have a significant therapeutic impact on each of the chronic conditions associated with insulin resistance without the past toxicity problems associated with the use of TZDs (151).

### The Resolution Response as Systems-Based Biology

**Figure 8:**
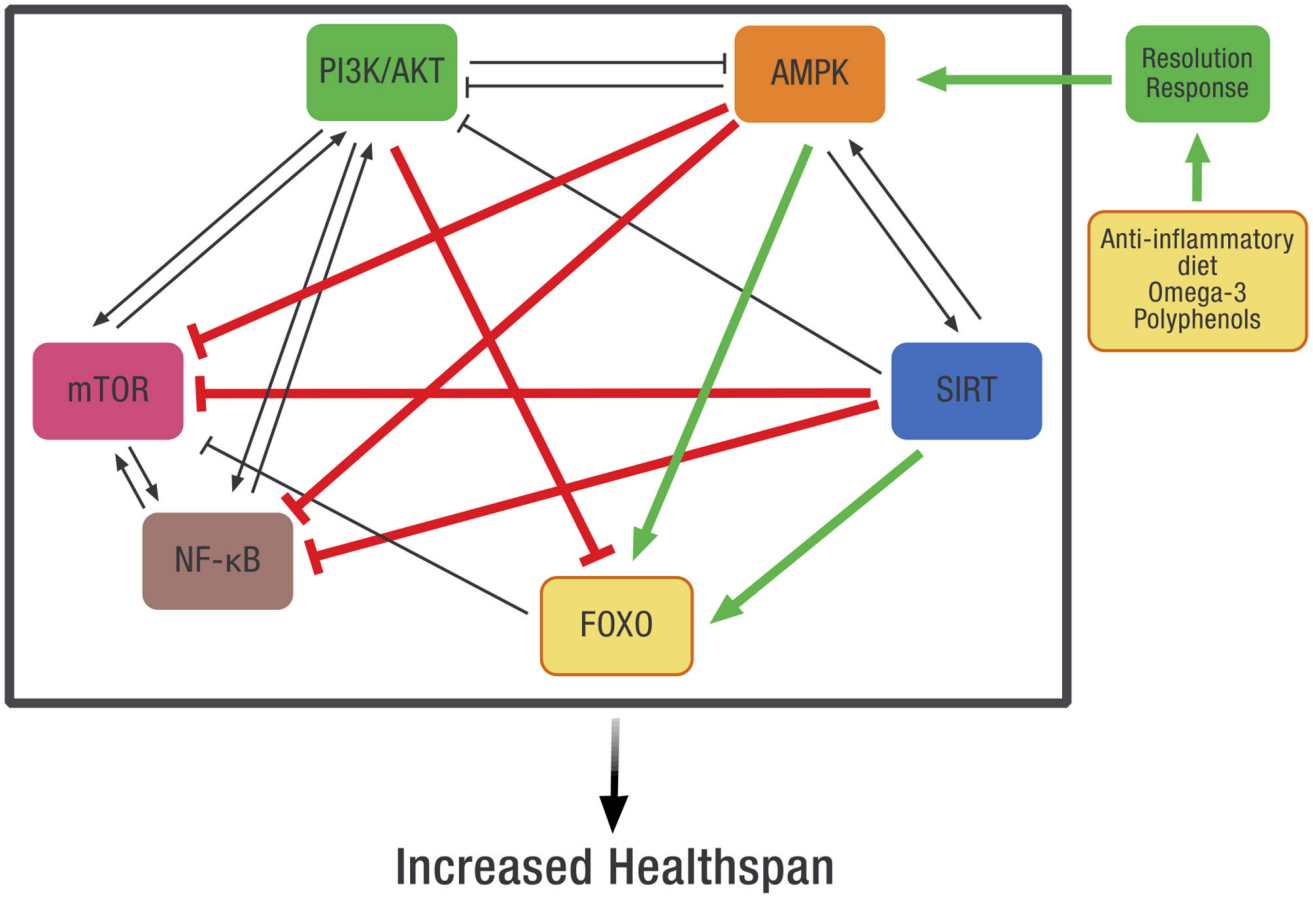
The potential effect of dietary optimization of the Resolution Response on various regulatory proteins and gene transcription factors. AMPK, 5' adenosine monophosphate-activated protein kinase; FOXO, Forkhead box transcription factors class O; mTOR, mammalian target of rapamycin; NF-κB, Nuclear factor kappa-B; PI3K/AKT, Phosphatidylinositol 3-kinase/AKT (protein kinase B).

Systems-based biology takes into account the interconnected signaling pathways within the cell that are required to maintain homeostasis. Because of these complex relationships, pharmaceutical intervention in one pathway may adversely affect other intracellular pathways. Our working hypothesis is that dietary optimization of the Resolution Response will result in the activation of AMPK that can more effectively coordinate these pathways. Although direct measurement of AMPK activity in any tissue is complex because it requires a biopsy, one can use the clinical blood markers described earlier that define optimization of the Resolution Response as surrogate markers for the maintenance of intracellular AMPK activity. How AMPK activity is intimately connected to many of these diverse intracellular signaling pathways is shown in Figure 8.

As shown in Figure 8, there is significant cross-signaling between these various metabolic systems within the cell and potential inhibition or activation of one system by another. In some cases, there is mutual activation, such as between AMPK and SIRT or between PI3K/AKT, mTOR, and NF-κB. In other cases, there can be reciprocal inhibition between systems, such as between PI3K/AKT and AMPK. Finally, there can also be unidirectional inhibition or activation between various signaling pathways.

AMPK may represent the molecular link between these diverse signaling systems and the diet. This control is possible since AMPK is an evolutionarily conserved energy sensor that controls metabolism. In essence, AMPK becomes the checkpoint for metabolic control that links diet to these various intracellular signaling systems. In particular, activation of AMPK will inhibit the activity of NF-κB, thereby reducing the generation of pro-inflammatory mediators such as cytokines and eicosanoids (48), but also inhibit other intracellular systems such as mTOR and the PI3K/AKT that can activate NF-κB (152). In addition, the repair of damaged tissue also requires increased AMPK activity for both the repair of damaged tissue and the prevention of fibrosis (153, 154).

However, one can only routinely monitor the blood, not the interior of the cell. This is why constant monitoring of the blood markers that define the Resolution Response provides an easily obtained insight into AMPK activity. Thus, continuous dietary optimization of the Resolution Response results in the dietary control intracellular AMPK activity. In doing so, it may be possible to maintain these other internal cellular signaling pathways within their optimal operating parameters. The successful result leads to improved metabolic efficacy generating a potentially extended healthspan.

On the other hand, any reduction in AMPK activity leads to the over-expression of pro-inflammatory signaling systems shown in Figure 8. One of the linked systems that would increase with a decrease in AMPK activity would include NF-κB. Increased NF-κB activity is related to increased inflammatory activity associated with cardiovascular disease (155) and cancer (156). Likewise, reduced activity of AMPK would lead to potentially excessive activity of mTOR and the PI3K/AKT signaling pathways associated with cancer (157, 158).

How the activity of AMPK acts as the central hub linking various other cellular signaling systems at the molecular level is described in more detail in the following summaries.

### NF-κB Signaling

One of the primary benefits of activating AMPK is the inhibition of NF-κB, resulting in the reduction of cytokine and eicosanoid formation. The lowering of inflammation is achieved through several different routes orchestrated by AMPK (48). One pathway is inhibiting NF-κB by the direct activation of AMPK (159). Another route is through the activation of SIRT1 by increasing NAD^+^ levels (63). Finally, AMPK activates the rate-limiting enzyme in the NAD^+^ salvage pathway that provides the necessary NAD^+^ to enable SIRT1 to deacetylate the Rel/p65 component of NF-κB to prevent its binding to the cell's DNA that is required to express inflammatory mediators (160).

Additional AMPK-mediated pathways that inhibit NF-κB activity include the activation of PGC-1α (161) and the phosphorylation of FOXO (49).

### mTOR Signaling

Activation of AMPK is the primary inhibitor of mTOR. At the molecular level, mTOR inhibition is due to phosphorylation of the raptor component of mTORC1 and TSC2 (162). In addition, the association of increased SIRT1 activity with the inhibition of mTOR (163) can be induced by the AMPK's activation of the rate-eliminating enzyme of the NAD+ salvage pathway (161). On the other hand, any increase in AKT activity will up-regulate mTOR, which activates NF-κB (164).

### PI3K/AKT Signaling

The PI3K/AKT pathway is activated by insulin and results in cellular growth activation (165). If the PI3K/AKT pathway is too active, this will inhibit the activity of AMPK (166, 167). On the other hand, any increase in AMPK activation will inhibit AKT activity (166, 167).

The inhibition of AMPK and FOXO activity by AKT can be reduced by optimization of the Resolution Response. One way in which PI3K activity can be reduced by lowering blood insulin levels following an anti-inflammatory diet. The reduction of PI3K activity leads to decreased activation of AKT. Long-term studies using the previously described anti-inflammatory diet have demonstrated success in the long-term management of type 2 diabetes (168, 169).

### FOXO Signaling

The FOXO family of gene transcription factors consisting of FOXO1, FOXO3, FOXO4, and FOXO6. The FOXO family is vital in controlling cellular senescence, stem cell maintenance, and lifespan in animal models (170). FOXO upregulation can be achieved either by phosphorylation *via* AMPK or deacetylation by SIRT (171, 172). In addition to the direct effect of AMPK activation on FOXO, any increase in AMPK activity will increase the activity of the rate-limiting enzyme (NAMPT) in the synthesis of NAD^+^, thereby activating SIRT, which also increases FOXO activity (173).

An indirect route to activate FOXO is *via* the AMPK-induced inhibition of AKT (171). On the other hand, any up-regulation of AKT by a deficit in AMPK activity will reduce FOXO activity (174–176). This central role of AMPK in FOXO activation may explain why activation of AMPK has been hypothesized to control the aging process (177).

Another inflammatory pathway that can be modulated by AMPK is JAK-STAT, which mediates cytokine signaling (178).

Considering the complexity of these interactions with cellular signaling mechanisms in the cell, optimizing the Resolution Response may have a far greater potential to bring a cell back to homeostasis than any potential drug therapy proposed for healthspan extension (179). If so, then the continuous optimization of the Resolution Response by the diet may play an essential role in extending the human healthspan.

### Limitations and Outstanding Questions

Our working hypothesis is that gaining control of the complex systems biology that occurs inside a cell will require consistent maintenance of AMPK within defined operating parameters to respond to constantly changing metabolic needs. Furthermore, we feel no single dietary intervention will provide that necessary control of AMPK to be used as a therapeutic intervention to treat the various chronic conditions associated with insulin resistance. While there is suggestive evidence that each dietary component of the Resolution Response (an anti-inflammatory calorie-restricted diet, adequate intake of omega-3 fatty acids and polyphenols) can have some health benefits in humans, we believe that their synergistic interactions will result in far more significant clinical benefits potentially mediated by their synergistic interactions on AMPK activity.

One major limitation of our hypothesis is the difficulty of measuring AMPK activity in humans as it requires a tissue biopsy. On the other hand, we feel the dietary optimization of the Resolution Response using defined blood markers can be titrated to their appropriate ranges results allows the development of the necessary clinical trials combining all three dietary interventions being applied simultaneously can be undertaken today.

Another limitation is the potential genetic variations between individuals. However, this can be overcome by titration of the dietary components of the Resolution Response to reach the appropriate target ranges in the blood. In essence, one would be undertaking an “AMPK” clamp to determine if the clinical condition being studied responds with equal, if not superior, clinical results to this type of dietary therapy compared to standard drug therapy.

To answer the question of the widespread clinical utility of our hypothesis requires doing clinical trials. Since one is using dietary interventions, clinical trials to optimize the Resolution Response bypass the need for animal models. As demonstrated by the CALERIE studies, calorie restriction can be maintained for an extended period of time (6, 7). Using omega-3 fatty acids and polyphenols at levels that have Generally Regarded as Safe (GRAS) status set the upper limits for their supplementation to a calorie-restricted anti-inflammatory diet with a defined protein-carbohydrate ratio. The most likely chronic conditions that would be applicable to such immediate clinical trials are those associated with insulin resistance with defined clinical endpoints such as metabolic syndrome, type 2 diabetes, and non-alcoholic fatty liver disease. If successful, then the same dietary technology should be applicable to other chronic diseases associated with insulin resistance.

## Summary

We hypothesize that the ability to heal from any injury-induced inflammation depends significantly on the dietary control of the body's internal Resolution Response. The final result of the optimization of the Resolution Response is the activation of AMPK. This hypothesis is based on the ability of AMPK to modulate internal cellular signaling through systems-based biology. While there is no single specific nutrient to optimize the body's internal capacity to heal from injury-induced inflammation, an appropriate combination of dietary interventions can alter signaling pathways that can lead to the molecular goal of increasing AMPK activity. This concept of requiring a defined combination of multiple dietary interventions to achieve the appropriate activation of AMPK is no different from using various combinations of discrete chemotherapeutic drugs to treat cancer. However, unlike numerous combination drug therapies used for cancer treatment, each dietary intervention described earlier can be easily modulated using the clinical markers that define the boundaries that optimize the Resolution Response for dietary guidance. Thus, the use of blood markers becomes the foundation for precision nutrition.

In conclusion, we believe understanding the complex interaction of highly defined dietary interventions that result in the optimization of the Resolution Response can provide a new appreciation of a new comprehensive nutritional strategy to treat many chronic conditions, especially those associated with insulin resistance. Furthermore, the dietary approach we have outlined can be optimized on an individual basis using validated blood markers to orchestrate a wide variety of internal cellular signaling systems. By using such blood markers to titrate each dietary component of the Resolution Response to their appropriate ranges moves precision nutrition into the realm of personalized medicine. Reaching and maintaining the appropriate ranges of those clinical markers that define the optimization of the Resolution Response may potentially be translated into an increased healthspan using diet *as if* it were a drug that controls the complex systems-based biology of healing.

## Author Contributions

BS and AS wrote the paper. All authors contributed to the article and approved the submitted version.

## Conflict of Interest

BS and AS are employed by Zone Labs, Inc., a medical foods company.

## Publisher's Note

All claims expressed in this article are solely those of the authors and do not necessarily represent those of their affiliated organizations, or those of the publisher, the editors and the reviewers. Any product that may be evaluated in this article, or claim that may be made by its manufacturer, is not guaranteed or endorsed by the publisher.

## Glossary

AA, Arachidonic acid; ACC 1 and ACC2, Acetyl-CoA carboxylase 1 and 2; AGE, Advanced glycosylated end products; AMPK, 5' adenosine monophosphate-activated protein kinase; APC, antigen-presenting cell; COX-2, Cyclooxygenase-2; DAMP, Danger-associated molecular patterns; DHA, Docosahexaenoic acid; DPA, Docosapentaenoic acid; ECM, Extracellular matrix; EPA, Eicosapentaenoic acid; FOXO, Forkhead box transcription factors class O; GLP-1, Glucagon-like peptide; Glut, Glucose transporter protein; GS, Glycogen synthetase; HbA1c, Hemoglobin A1c; JAK, Janus kinase; LKB1, Liver kinase B1; mTOR, Mammalian target of rapamycin; NAMPT, Nicotinamide phosphoribosyltransferase; NF-κB, Nuclear factor kappa-B; NOS, Nitrogen oxide synthetase; PYY, Peptide YY; PGC-1α, Peroxisome proliferator-activated receptor gamma coactivator 1-alpha; RAGE, Receptor for advanced glycation end products; RvD1, Resolvin D1; SASP, Senescence associated secretory phenotype; SCFA, Short-chain fatty acids; SIRT, Sirtuins (silent information regulator family of proteins); SPMs, Specialized pro-resolving meditators; SREBP-1c, Sterol regulatory element-binding protein-1c; STAT, Signal transducer and activator of transcription; TNFα, Tumor necrosis factor-alpha; TLR-2, Toll-like receptor-2; TLR-4, Toll-like receptor-4; TSC2, Tuberous sclerosis complex 2; ULK-1, Unc-51-like kinase 1.
